# Discerning Amyloid‑β and Tau Pathologies with Learning-Based Quantum Sensing

**DOI:** 10.1021/acsphotonics.5c01192

**Published:** 2025-09-25

**Authors:** Shruti Sundar, Marakkarakath Vadakkepurayil Jabir, Lukas Glandorf, Maria Eleni Karakatsani, Michael Reiss, Ruiqing Ni, Daniel Razansky

**Affiliations:** † Institute for Biomedical Engineering and Institute of Pharmacology and Toxicology, Faculty of Medicine, 27217 University of Zurich, Zurich 8057, Switzerland; ‡ Institute for Biomedical Engineering, Department of Information Technology and Electrical Engineering, 27219 ETH Zurich, Zurich 8093, Switzerland; § National Institute of Standards and Technology, Gaithersburg, Maryland 20899, United States

**Keywords:** Entanglement decoherence, spontaneous parametric down conversion, polarization entangled photons, supervised machine learning, Alzheimer’s disease

## Abstract

Photon entanglement, a key feature of quantum correlations, provides a level of coherence absent in classical correlations, potentially offering new information when interacting with biological matter. One promising application is using entanglement decoherence to distinguish between healthy and diseased samples. However, achieving this requires efficient entangled photon sources capable of surviving through biological samples for reliable detection. In this work, we show the applicability of a polarization-entangled photon source as a label-free diagnostic tool for distinguishing between transgenic mouse models of amyloidosis and tauopathy and their respective control strains. We investigated cortical and hippocampal regions of these models, and our findings revealed greater preservation of entanglement in the transgenic samples compared to controls. To further enhance classification accuracy, we employed a supervised machine learning approach, achieving reliable distinctions between disease and control groups in unseen test samples. The quantum-based results were further validated through confocal imaging of the transgenic and control samples. These findings suggest that quantum sensing could serve as a label-free approach for distinguishing biological samples, with potential applications in the study of neurodegenerative disorders.

## Graphical abstract

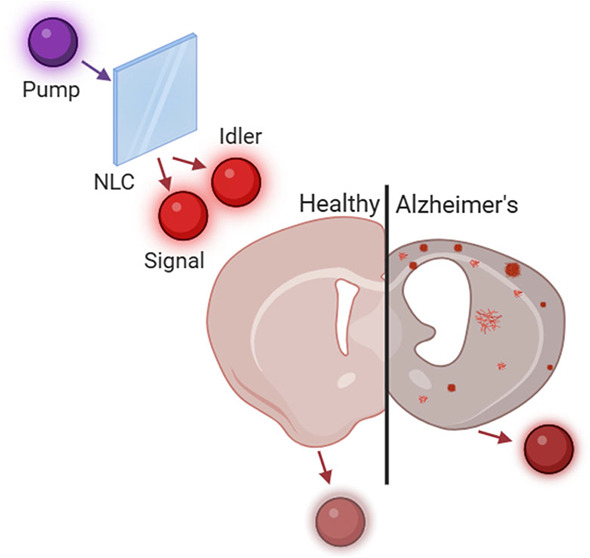

## Introduction

Alzheimer’s disease (AD) is a chronic, progressive neurodegenerative disorder that predominantly affects memory, cognition, and behavior.^1^ The hallmark of AD is the accumulation of two abnormal protein aggregates in the brain, accompanied by neuronal cell loss, primarily affecting the hippocampus and cortex: amyloid-β (Aβ) plaques, which are misfolded amyloid-β deposits that accumulate extracellularly, and neurofibrillary tangles, composed of intracellular aggregates of hyperphosphorylated tau protein.^2,3^ These pathological features are associated with widespread neuronal damage, cognitive decline, and, ultimately, loss of independence in affected individuals. Clinical diagnostics for AD typically involves measuring regional brain atrophy using structural MRI, PET imaging of glucose metabolism with fluorodeoxyglucose (FDG), and amyloid, as well as invasive cerebrospinal fluid (CSF) sampling to measure tau and amyloid levels.^4–7^ However, functional changes at the molecular level, undetectable by MRI, often occur before brain atrophy and may precede any cognitive symptoms. While PET imaging is effective, it is an expensive modality that relies on toxic radionuclides, and CSF sampling is a painful and invasive procedure. The gold standard for diagnosing AD remains the neuropathological examination of post-mortem brain tissue, detecting Aβ plaques and tau protein tangles using immunohistochemical techniques. Various exogenous reagents, including fluorescent dyes such as Congo red and Thioflavin S, along with antibodies, are used for biomarker visualization in *ex vivo* and *in vitro* studies of AD.^8^ However, histological analysis involves a labor-intensive process, including brain fixation, tissue processing, sectioning, and staining before microscopic examination. This protocol is costly, time-consuming, and highly dependent on the pathologist’s expertise. Therefore, there is a need for a label-free, less labor-intensive technique.

A number of techniques based on optical microscopy have shown promising results in label-free visualization of senile plaques in thin slices of fixed brain tissue. These include optical coherence microscopy (OCM), polarization-sensitive optical coherence microscopy (PS-OCM), cryo-micro-optical sectioning tomography (cryo-MOST), stimulated Raman scattering (SRS) microscopy, multiphoton microscopy.^9–14^ However, these techniques rely on expensive equipment, complex configurations, and precise scanning systems. Polarized light-based imaging and characterization techniques have long been used in biomedical research.^15^ Scattering alters the degree of polarization and the state of polarization of the incident light beam.^16^ Mathematical formalisms dealing with propagation of polarized light and its interaction with any optical system can be described by two formalisms; the Jones calculus which is a field-based representation and the Stokes–Mueller calculus that is an intensity-based representation. A major drawback of the Jones formalism is that it deals with pure polarization states only and cannot handle partial polarizations and therefore depolarizing interactions which are common in biological tissues and thus is used for clear and nondepolarising media such as thin films only. Stokes–Mueller polarimetry can handle partially polarized and depolarizing tissues, yet still relies primarily on intensity-based measurements, potentially limiting sensitivity to subtle structural changes.^17,18^ Additionally, theoretical and experimental efforts have been made toward studying the propagation of classically entangled and quantum entangled source propagating through biological samples and scattering solution.^19–27^ Quantum sensing offers distinctive advantages that classical methods cannot provide. Quantum correlations inherent in entangled photon pairs are sensitive to subtle scattering-induced depolarization quantified as decoherence. This intrinsic quantum sensitivity allows direct, label-free detection of minute structural changes in tissues, such as those caused by Alzheimer’s disease pathology, without relying on external contrast agents. Previous work employing quantum sources demonstrated promising initial results in differentiating pathological from healthy tissues.^22,28^ However, these studies were limited by low photon flux, limited pair generation rates, and the requirement for temporal compensation, which collectively diminished the signal-to-noise ratio (SNR), prolonged acquisition times, and reduced practical applicability. Additionally, the absence of comprehensive examinations across different brain regions and pathologies, the limited use of advanced classification techniques, and the lack of validation with conventional imaging methods have constrained the diagnostic reliability and overall scope of these studies.

In this work, we employ a home-built polarization-entangled photon source that requires no phase or temporal compensation and offers a high pair generation rate. This design enables short integration times per measurement, thereby reducing the overall acquisition time. Moreover, the high photon flux and the use of a narrow coincidence window minimize accidental coincidences, resulting in an improved coincidence-to-accidental ratio (CAR). The entangled photons are generated at room temperature and can be transformed into different Bell states using phase plates. We employed our entangled photon source to investigate the decoherence of quantum entanglement in Bell states across various mouse models. Specifically, we used two transgenic mouse models, an amyloidosis model (5xFAD) and a tauopathy model (P301L), along with their respective control strains (CD1 and NTL), with four mice per model. These models reliably replicate key pathological features relevant to human Alzheimer’s disease. Using two distinct transgenic models allowed us to examine the separate effects of tau and amyloid-β on entanglement parameters, while including two control strains ensured robust comparisons and accounted for potential strain-specific variations. Data from three mouse brain samples were used to train a supervised machine learning model to establish diagnostic thresholds, which were subsequently applied to classify unknown samples from the same models, demonstrating reliable diagnostic performance. Furthermore, we validated our findings in both transgenic and control samples using immunohistochemical techniques and a commercial confocal microscope.

## Results

### Methodology for Distinguishing *Ex Vivo* Amyloidosis and Tauopathy Mouse Brain Samples *via* Quantum Sensing

Figure 1a shows the schematic of the polarization-entangled photon source. A continuous-wave, single-frequency 405 nm laser (Obis, Coherent Inc., Saxonburg, PA, USA) served as the pump source. The beam was directed into a 20 mm long, 2 × 1 mm^2^ aperture PPKTP (Periodically Poled Potassium Titanyl Phosphate) crystal, with a grating period of Λ = 3.425 μm (Raicol Crystals, Rosh Haayin, Israel), to achieve type-0 quasi-phase-matched down-conversion of the 405 nm pump beam. A convex lens (*f* = 250 mm) focused the beam at the center of the crystal. The crystal was placed in a temperature controller at the center of a Sagnac interferometer.^29,30^ The interferometer consisted of a dual-wavelength polarizing beam splitter (D-PBS), a dual-wavelength half-wave plate (D-HWP), and two high-reflectivity broadband mirrors (*R* > 99%), M1 and M2. This setup generated noncollinear, degenerate, entangled photon pairs at 810 nm. The photons generated by spontaneous parametric down-conversion (SPDC) in both clockwise and counterclockwise directions were combined at the DPBS. The two-photon polarization state at the output ports of the interferometer is expressed as follows

1 $$\left|\Phi^{\pm }\right\rangle =\frac{|H_{s}H_{i}\rangle \pm e^{\mathrm{i\varphi}}\left|V_{s}V_{i}\right\rangle }{\sqrt{2}}$$

where the subscripts s and i denote signal and idler, respectively. The phase factor φ arises from the relative phase difference between the two photon amplitudes. This phase shift was compensated by positioning the crystal at the center of the Sagnac interferometer on a linear stage. The PPKTP crystal was placed symmetrically relative to the DPBS, making the experimental setup robust against optical path changes.^29^ A coated silver right-angled prism mirror splits the entangled photons into two paths: the heralded path and the sample path. In the sample path, photons were focused onto the sample, which was mounted between a glass slide and a coverslip on a 3-axis translational stage. The entangled photons were focused with a 10× objective, collimated with another 10× objective, and passed through the analyzer, comprising a quarter-wave plate (QWP), half-wave plate (HWP), and polarizer, before being coupled into a single-mode fiber. The Gaussian waist at the sample was ∼20 μm, with a Rayleigh range of 1.5 mm. In the heralded path, photons passed through the analyzer optics (QWP, HWP, and polarizer) before being coupled into a single-mode fiber. An HWP (HT) was inserted into one path, and a QWP (QT) was placed in the other path to enable transformation between Bell states. Interference filters with a 10 nm transmission bandwidth, centered at 810 nm, were added to both paths. The entangled photons were detected using single-photon avalanche detectors (Si-APDs, ID120, ID Quantique SA, Geneva, Switzerland), and the photon pairs were further analyzed with a coincidence counter (ID1000, ID Quantique). At a pump power of 5 mW, and a 10 ns coincidence window, we measured a coincidence count rate of 7650 Hz, corresponding to an estimated pair generation rate of approximately 13,600 Hz after efficiency correction. With single count rates of ∼80 kHz, the accidental coincidence rate is approximately 64 Hz, ensuring a high coincidence-to-accidental ratio and robust signal fidelity. Throughout the study, we employed a pump power of 5 mW, a 10 ns coincidence window, and a 1-s integration time per tomographic projection measurement. In contrast, other studies, limited by lower photon pair generation rates, required integration times ranging from 18 to 150 s per tomographic measurement.^22,28^ Consequently, their total tomographic reconstruction times ranged from 4.8 to 40 min for 16 measurements, with a longer 40 ns coincidence window. In our study, the shorter integration time was made possible by the higher pair generation rate, and the reduced noise by the narrower coincidence window. While manual adjustments were needed to rotate a single waveplate between measurements, these were performed quickly and did not significantly affect the total acquisition time, which was primarily governed by the 1-s integration time for each tomographic measurement. All samples were 40 μm thick. Brain samples were collected from four mouse models: P301L (tauopathy model), with a nontransgenic littermate (NTL) as its control, and 5xFAD (amyloidosis model), with CD1 as its control. For each strain, samples were obtained from four individual mice (both sexes), with P301L and NTL mice aged 10–12 months and 5xFAD and CD1 mice aged 5–6 months. This approach ensured sufficient biological variability for statistical comparisons between transgenic and control brain tissue. We used two different control strains, NTL and CD1, as each is conventionally paired with its respective transgenic models (P301L and 5xFAD), allowing for more relevant comparisons within each disease pathway. Additional information regarding the animal models can be found in the Methods section under ‘Animal Models’. The quantum source was focused on the cortex and hippocampus, regions critically affected by Alzheimer’s.^31^

**Figure 1:**
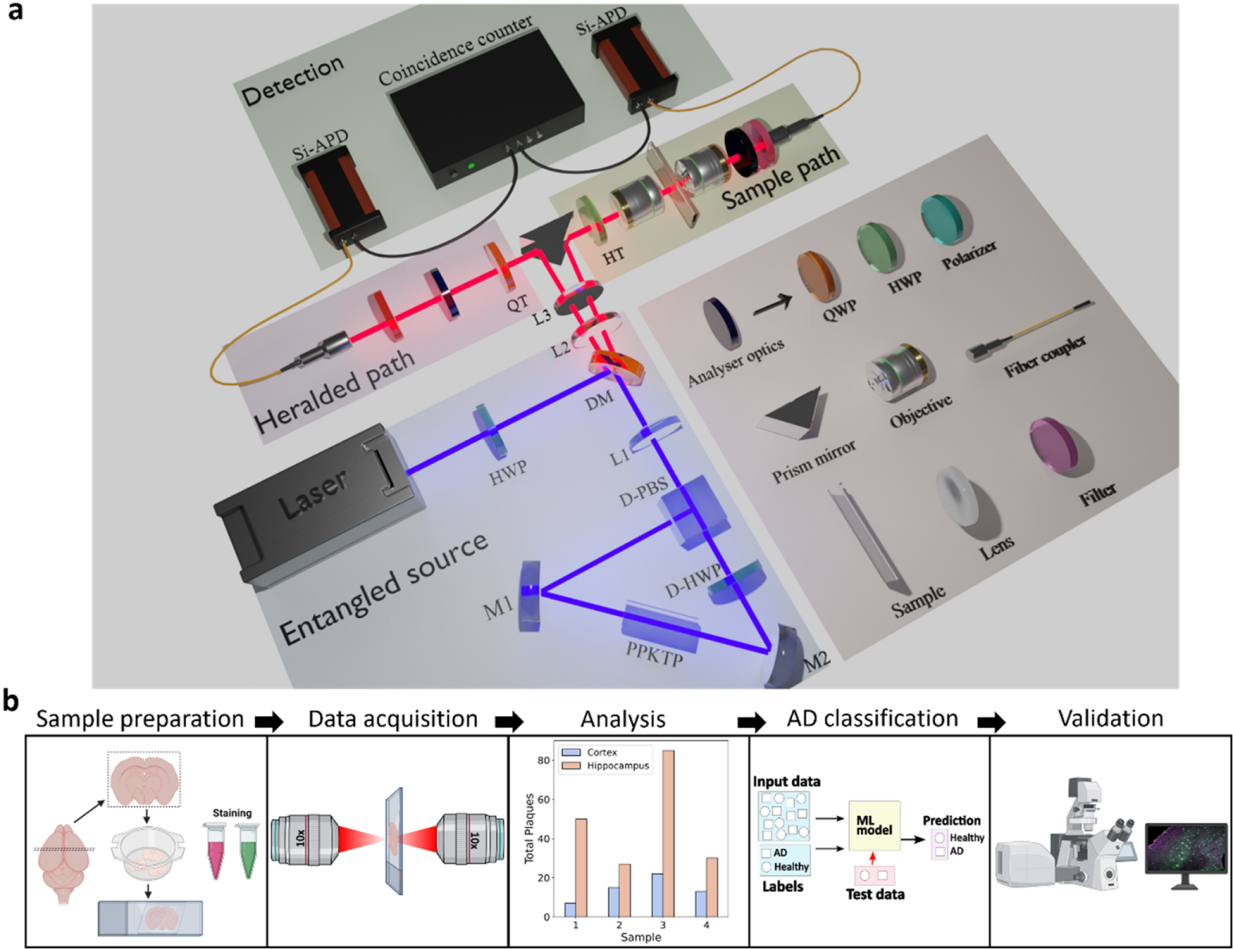
Overview of the experimental setup and workflow for studying AD using a quantum entanglement-based sensing approach. (a) Schematic of the polarization-entangled photon source with a type-0 PPKTP crystal inside a temperature controller, placed in a Sagnac loop. M1, M2: mirrors; L1–L3: lenses; D-PBS: dual-wavelength polarizing beam splitter; D-HWP: dual-wavelength half-wave plate; HWP: half-wave plate; DM: Dichroic mirror; HT, QT: half-wave plate and quarter-wave plate for transformation into different Bell states, respectively. The analyzer consists of a quarter-wave plate, a half-wave plate, and a polarizer. Si-APD: silicon-based single-photon avalanche detectors. (b) Workflow of the experiment, detailing sample preparation, data acquisition with quantum approach, data analysis, AD classification using supervised machine learning, and correlation with a commercialized confocal microscope.

Figure 1b shows the workflow of this experiment, which begins with the preparation of the sample by sectioning the whole brain into 40 μm-thick slices and staining with antibodies. Antibody staining is solely used for reference measurements. The samples were probed using the entangled photon source, which involves performing quantum state tomography of the entangled photon state that passes through different brain regions and calculating entanglement parameters. This data was used to train a machine learning model, specifically a support vector machine (SVM), to distinguish control (NTL, CD1) and pathology-bearing (P301L, 5xFAD) samples. Finally, the observations from the quantum source were validated with results obtained from a commercial confocal system. Detailed information on sample preparation can be found in the Methods section under ‘immunofluorescence staining’.

### Reconstruction of Density Matrix of the |Φ+⟩ State in Different Mouse Models

Quantum entanglement provides a high degree of coherence between correlated particles, which can evolve coherently among entangled particles traveling separate paths and affect each other in the correlation when one of them is measured. As the light travels through a scattering medium, it gradually loses its coherence. However, environmental decoherence effects transform a pure entangled state into a statistical mixture, degrading quantum entanglement as it passes through a scattering medium. This gradual decoherence provides the potential for distinguishing between tissue types.^28^ Werner states are important in quantum information theory as they represent a class of mixed quantum states that can exhibit either entanglement or separability, depending on their parameters.^32,33^ These states are crucial for investigating the boundary between classical and quantum correlations and for studying the robustness of entanglement in the presence of decoherence or quantum operations. When light prepared in an entangled state passes through a scattering medium, it undergoes a transformation, evolving into a state that lies between a maximally entangled and a maximally mixed state, which is consistent with the characteristics of a Werner state. A Werner state takes the following form of a density matrix

2 $$\rho_{w}=p\left|\phi \right\rangle \left\langle \phi \right|+\frac{I\left(1-p\right)}{4}$$

Where |ϕ⟩ is the maximally entangled state, *i.e.*, Bell state, *I* is the identity matrix, and *p* is the Werner probability.^33^

During acquisition, one of the photons of a pair was transmitted through the brain sample (cortex and hippocampal regions) and the subsequent state analyzer, while the other photon of the pair travels only through the state analyzer before reaching to Si-APDs through a single-mode fiber. The field of view (FOV) is limited to the focal spot where entangled photons were focused through the brain sample, covering an approximate region of 20 μm based on the objective’s beam waist. The polarization entangled state analyzer were used to effectively project the polarization state of the light onto any of the six main polarization eigenstates: linear horizontal, linear vertical, diagonal (+45° relative to horizontal), antidiagonal (−45° relative to horizontal), as well as right and left circular states. We used a standard quantum tomographic technique of 16 polarization-projective measurements, to determine the density matrix of the light. QT and HT were then used to transform to a different Bell state.^34^ Figure 2a shows the real part of the reconstructed density matrix when the |Φ+⟩ state passes through the hippocampus of one sample of P301L and NTL, respectively. The P301L sample shows much lower decoherence as compared to the NTL. Similarly, the 5xFAD sample exhibits nearly no decoherence compared to the CD1 (Figure 2b). This finding suggests that entanglement decoherence is generally lower in transgenic models than in their respective controls samples. Furthermore, the hippocampus region of the 5xFAD sample preserves entanglement more effectively than the P301L sample. More information regarding state reconstructed from the rest of the Bell states is provided in the supplementary document (Figure S1).

**Figure 2:**
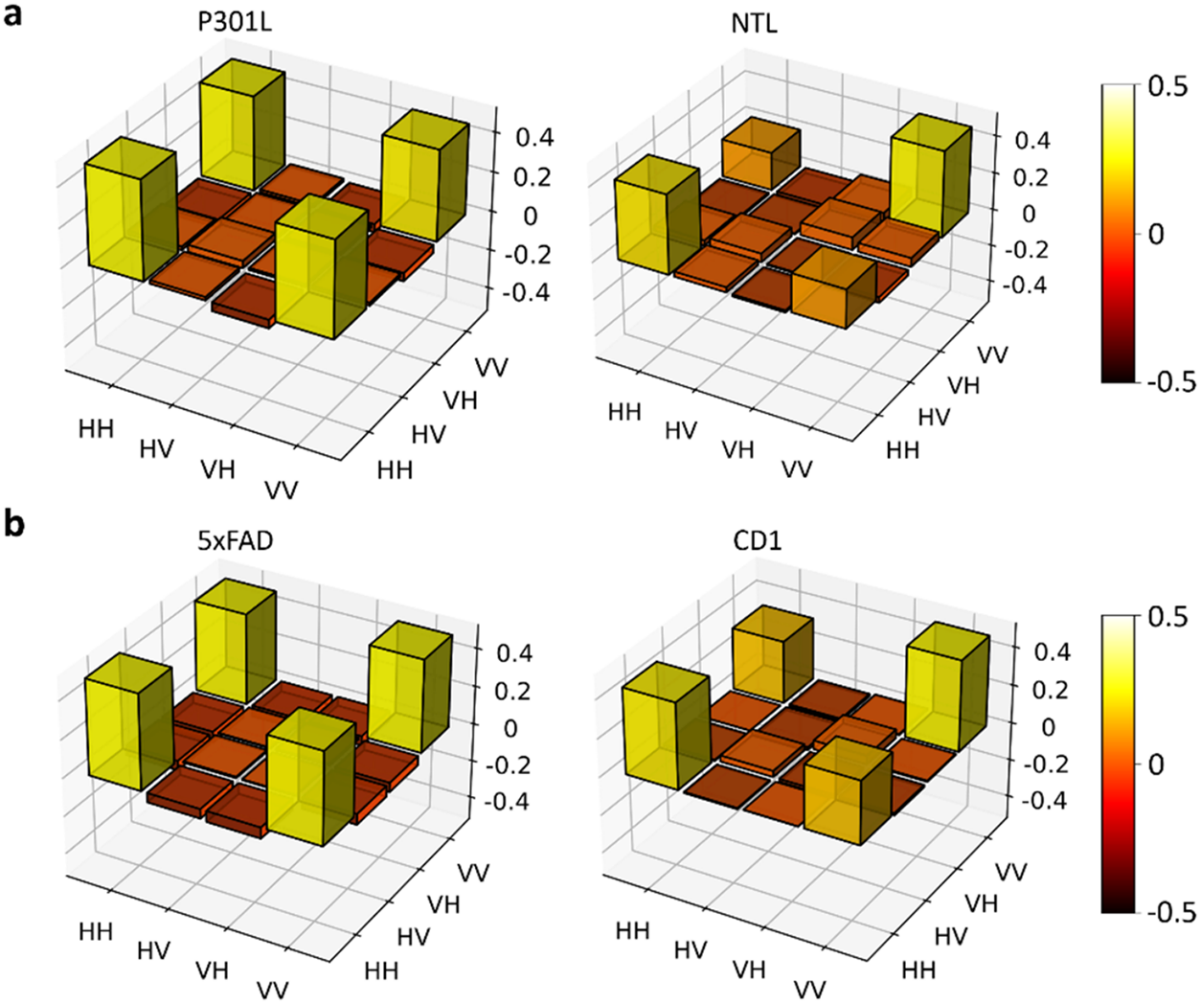
Reconstructed density matrix of the source after |Φ+⟩ state transmits through four mouse models in the hippocampus. Experimental results of the tomographic measurements for (a) P301L and NTL sample. The bar graph shows the real part of the reconstructed density matrices of the light after one photon passed through either the P301L or NTL sample. Tomography was performed using coincidence events from 16 measurement sets in both cases. (b) 5xFAD and CD1 sample, shown with the same tomographic procedure and bar graph layout as in (a).

### Distinguishing Samples Based on Entanglement Decoherence

Based on the tomographic reconstruction described in the Methods section, entanglement parameters such as tangle, and linear entropy were calculated. We focused on the tangle (T) and the linear entropy (S) as they characterize distinct aspects of the entanglement state: with nonseparability quantified by T and coherence by S. The former specifies the degree of entanglement, ranging from 0 (separable states) to 1 (maximally nonseparable states); the latter specifies the degree of mixture, ranging from 0 (pure states) to 1 (maximal mixed states). It was previously found that entangled photons passing through a scattering medium degrade the entanglement along the Werner-state path, converting entangled photon pairs into incoherent pairs.^22^ Figure 3 shows the plot for T vs S with the Werner path shown in a solid black line. The blue marker on the plots (Figure 3a–d) represents the mean of five measurements, with error bars showing the standard deviation, obtained as a reference when entangled photons were passed through the same spot of a glass slide. It exhibits minimal decoherence, as expected when passing through a transparent medium. Each red and green marker corresponds to the mean of five measurements from a single spot in the cortex (Figure 3a,c) or hippocampus (Figure 3b,d) of one transgenic sample (P301L or 5xFAD) and its respective control (NTL or CD1). Error bars indicate the standard deviation. Total acquisition time for each data point was ∼1.2 min. The diseased and healthy data deviate from the glass slide data, moving down the Werner curve, indicating that photons passing through the samples experience scattering, which leads to decoherence. This scattering, primarily caused by depolarization due to dephasing, reduces the entanglement of the quantum state, transitioning it from a purely entangled state to a partially mixed state. The data points follow the Werner state curve closely on the TS plot because scattering processes degrade entanglement without much photon loss. An example data set showing tangle variation as the ∼20 μm beam spot is moved across a region containing sparse Aβ plaques is provided in the supplementary file (Figure S2). The photons pass through both plaque-dense and plaque-sparse regions, with partial overlaps yielding intermediate entanglement values and near-complete overlap showing maximum preservation. Additionally, at 810 nm, water absorption is minimal, resulting in low photon loss and minimal deviation from the Werner curve. Figure 3a–d (right) shows confocal images of immunofluorescence-labeled brain samples from all four models, highlighting the cortical and hippocampal regions probed by the entangled photon source. NTL samples display the absence of tau pathology, whereas P301L samples show phosphorylated tau aggregates (green, AT-8, Alexa Fluor 488) with nuclei counterstained by DAPI (magenta). CD1 samples display c-Fos-stained neurons (magenta) in both cortex and hippocampus. In contrast, 5xFAD samples reveal amyloid plaques (green, 6E10, Alexa Fluor 488) alongside nuclei stained with DAPI (magenta). Notably, in our samples, the Aβ plaques appear more prominent in the hippocampus compared to the cortex.

**Figure 3:**
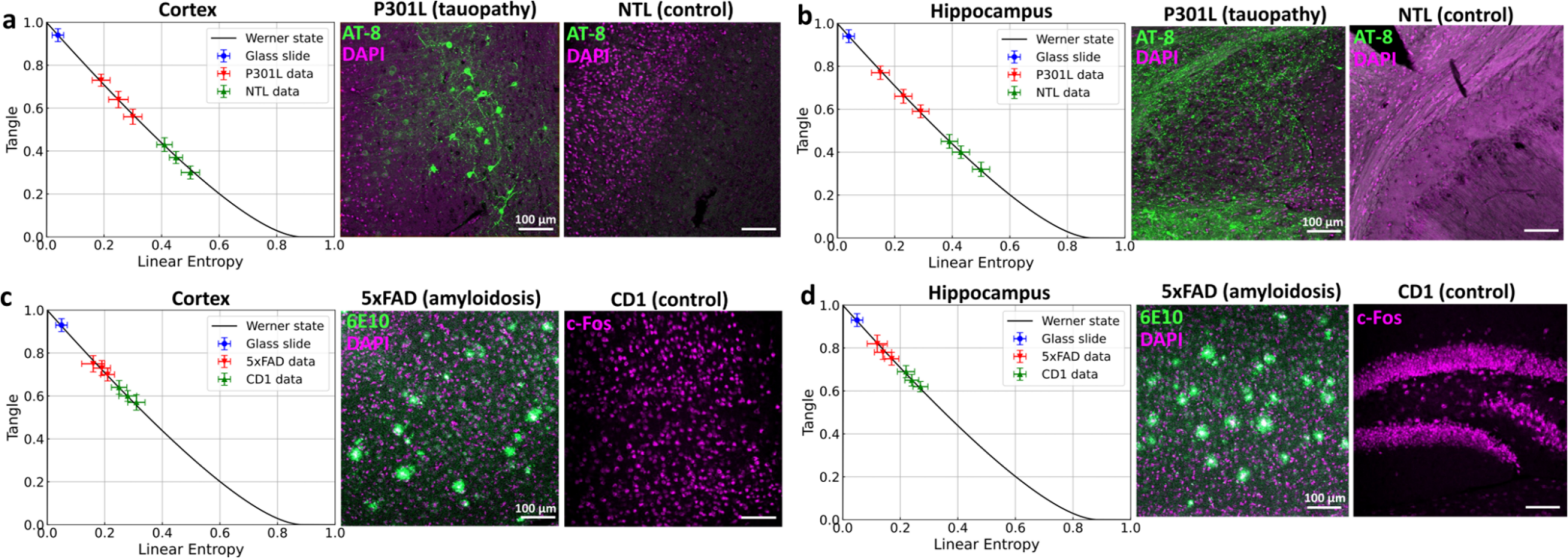
Comparison of entanglement decoherence across P301L–NTL and 5xFAD–CD1, using the Werner state model, measured with the |Φ+⟩ state. Decoherence in P301L–NTL samples in the cortex (a) and hippocampus (b). Decoherence in 5xFAD–CD1 samples from the cortex (c) and hippocampus (d). Each data point represents the mean of five repeated measurements at a single position, with error bars showing the standard deviation. The black line represents the theoretical Werner state curve. Confocal fluorescence images (right) with a field of view of 640 μm × 640 μm illustrate tau pathology (AT-8, green) in P301L and amyloid pathology (6E10, green) in 5xFAD, counterstained with DAPI (magenta). Controls (NTL, CD1) show absence of pathology, with CD1 stained for c-Fos (magenta). Measurements in the cortex and hippocampus were obtained from the same sample for each model. Scale bars: 100 μm.

From all the plots, it is evident that the P301L and 5xFAD samples exhibit less decoherence than the NTL and CD1, which may be attributed to the arrangement of scatterers in the brain tissue, potentially exhibiting translational invariance in one direction. Within each model (P301L and 5xFAD), we did not detect a significant difference between hippocampus and cortex for linear entropy (Wilcoxon signed-rank, P301L: *p* = 0.63 and 5xFAD: *p* = 0.25) or tangle (Wilcoxon signed-rank; P301L: *p* = 0.88, 5xFAD: *p* = 0.25). Although the confocal images (Figure 3c) qualitatively show regional variation in pathology load, these visual differences did not translate into significant differences in the entanglement metrics. Both models showed clear AD–control separation in cortex and hippocampus (Mann–Whitney U). In cortex, separation by tangle was larger for 5xFAD–CD1 (Hedges’ *g* = 8.27) than P301L–NTL (*g* = 6.17). In hippocampus, separation by tangle was larger for P301L–NTL (*g* = 8.09) than 5xFAD–CD1 (*g* = 5.15). Linear entropy effect sizes showed the same region-dependent pattern in absolute magnitude. This may suggest that the structural differences caused by Tau tangles have a more significant impact on the quantum entanglement properties than those caused by Aβ plaques, potentially offering better contrast for distinguishing the two conditions in our case. The P301L data also show increased scattering compared to the 5xFAD data in both regions, which could be influenced by natural sample variability or differences in water retention. Reduced water retention in the P301L–NTL sample might have contributed to higher scattering, resulting in increased linear entropy. The two healthy samples also show distinct properties, with one appearing more cluttered and the other more scattered. This discrepancy could stem from subtle differences in sample preparation, structural features or potential strain differences within the brain tissue itself. To better illustrate the sample variability, representative confocal fluorescence images from all biological samples across all mouse models for cortex and hippocampus are provided in supplementary file (Figures S3 and S4). More information regarding TS plots for the rest of the Bell states is provided in the Supporting file (Figure S5).

### Machine Learning-Assisted Classification of Brain Samples

To reliably distinguish between P301L, 5xFAD, and their respective control models, NTL and CD1, we employed a supervised machine learning approach using a support vector machine to classify previously untested brain samples. The SVM was chosen for its robustness in binary classification, its ability to find a clear decision boundary between the two groups based on entanglement parameters (tangle and linear entropy), and its interpretability, making it suitable for distinguishing group-specific differences. A SVM classifier with a linear kernel was trained to differentiate transgenic models from their respective controls using linear entropy and tangle values as input features. The SVM regularization parameter (*C*) was optimized using grid search with 5-fold cross-validation. No additional normalization or dimensionality reduction was applied, as features were inherently bounded (0–1) and limited to two clearly interpretable variables. We trained the model on three datasets (for example, samples 1, 2, and 3) that included all individual measurements from the samples and tested it on the fourth, unseen data set (for example, sample 4). This was repeated for each possible combination, ensuring that each data set served as an unseen test set once. The complete set of performance metrics (precision, recall, sensitivity, specificity, and F1-score) for each split in the hippocampal region for the 5xFAD and CD1 mouse models is provided in the supplementary file (Table S1). The SVM was trained separately for the cortex and hippocampus, and it determined an optimal hyperplane to differentiate between diseased and healthy samples. The model was then used to classify the unseen test data, with results visualized to show correctly classified and misclassified points for P301L–NTL cortex (Figure 4a), P301L–NTL hippocampus (Figure 4c), 5xFAD–CD1 cortex (Figure 4e), and 5xFAD–CD1 hippocampus (Figure 4g). Additionally, classifier performance was further quantified using precision, recall scores, sensitivity, specificity, and F1-score (Table 1). All metrics yielded a maximum value of 1, further confirming the SVM’s effectiveness in distinguishing between transgenic models and their respective controls. A comparison of 2D (linear entropy and tangle) *versus* 1D (Werner probability) SVM classification is provided in the supplementary file (Table S2 and Figure S6). Across data sets, the 2D SVM matched or outperformed the 1D approach, particularly when deviations from the Werner model carried discriminative information.

**Figure 4:**
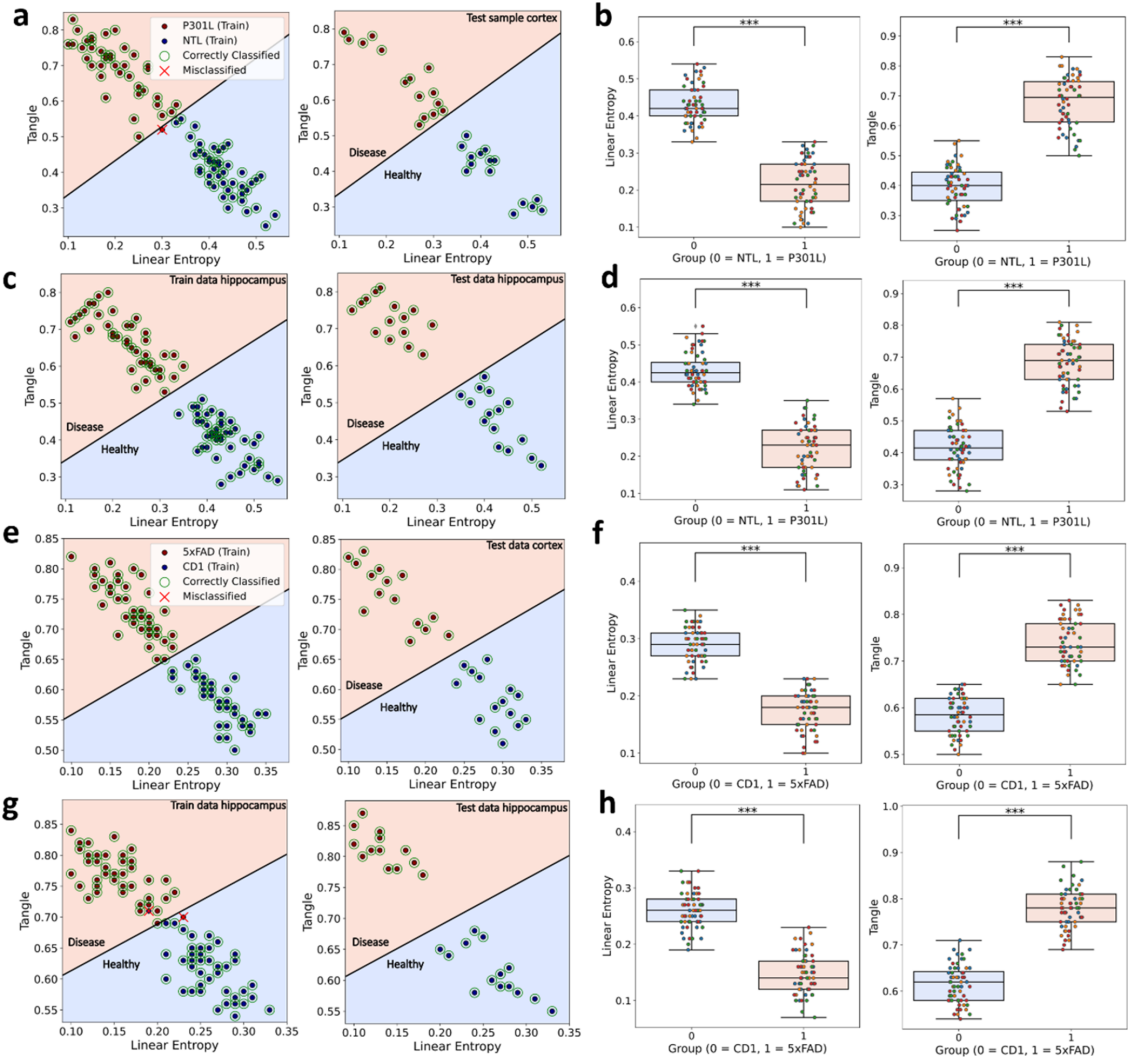
Support vector machine-based classification and statistical analysis of AD-affected and healthy brain samples in the cortex and hippocampus regions. SVM plots: (a) P301L–NTL cortex, (c) P301L–NTL hippocampus, (e) 5xFAD–CD1 cortex, (g) 5xFAD–CD1 hippocampus. Each plot shows the training and test data sets, highlighting correctly classified points and misclassified points. Box plots: (b) P301L–NTL cortex, (d) P301L–NTL hippocampus, (f) 5xFAD–CD1 cortex, (h) 5xFAD–CD1 hippocampus. Box plots represent the distribution of tangle and linear entropy values for each transgenic model (P301L, 5xFAD) and its respective control (NTL, CD1), comparing statistical differences between groups in each brain region. Each dot represents a single measurement, with colors indicating different biological replicates. The confidence level was set to 0.05 (*p*-value). The significance level is displayed as asterisks (***) *p* < 0.001.

**Table 1:** Complete SVM Classifier Performance Metrics

sample	region	precision	recall	sensitivity	specificity	F1-score
NTL	cortex	1	1		1	1
P301L		1	1	1		1
NTL	hippocampus	1	1		1	1
P301L		1	1	1		1
CD1	cortex	1	1		1	1
5xFAD		1	1	1		1
CD1	hippocampus	1	1		1	1
5xFAD		1	1	1		1

We performed Levene’s test to assess variance equality in tangle and linear entropy values between each transgenic model (P301L, 5xFAD) and its respective control (NTL, CD1) for both cortex and hippocampus. For groups with equal variances, we applied the two-sided *t* test; for those with unequal variances, we used the two-sided Mann–Whitney *U* test. In the cortex (Figure 4b) and hippocampus (Figure 4d) of P301L sample, significant differences were observed in linear entropy and tangle values (*p* < 0.001 for all cases). Similarly, in the cortex (Figure 4f) and hippocampus (Figure 4h) of 5xFAD samples, the *t* tests showed significant differences for both features (*p* < 0.001). Across all cases, effect sizes, measured by Cohen’s d or Glass’s delta, indicated large effects (|*d*| > 3.4), confirming a clear distinction between each transgenic model (P301L, 5xFAD) and its respective control (NTL, CD1). To quantify the precision of these effect sizes, we calculated 95% confidence intervals (CI), which provide a range within which the true effect size is likely to fall. For the P301L case, the 95% confidence intervals for tangle were (−4.50, −3.28) in both the cortex and hippocampus, while for linear entropy, the intervals were (−5.18, −3.82). In the 5xFAD case, the confidence intervals for tangle were (−4.33, −3.12) in the cortex, and (−4.50, −3.28) in the hippocampus. For linear entropy, the intervals were (−4.98, −3.65) in the cortex, and (−5.18, −3.82) in the hippocampus. The box-and-whisker plots illustrate a clear separation of tangle and linear entropy values between the two groups. All individual measurements from the four samples are shown, with different colors indicating the individual sample for each mouse model.

### Confocal Validation of Quantum Sensing Results

The confocal image analysis workflow, applied to a field of view of 640 μm × 640 μm in both the cortex (Figure 5a) and hippocampus (Figure 5b) of the 5xFAD model, enables quantitative assessment of the correlation between amyloid pathology and quantum state tomography results. Alexa Fluor 488 staining was used to specifically label plaques, which were then isolated from the raw data. Noise reduction was achieved using a median filter, followed by threshold-based segmentation to extract plaque regions. Subsequent processing steps included morphological hole filling and smoothing operations *via* erosion and dilation. The analysis involved counting plaques and calculating the percentage of plaque area defined as the ratio of total plaque area to total image area in both the cortex and hippocampus (Figure 5c). The plaque count and plaque area were higher in the hippocampus compared to the cortex. This also relates well with the fact that entanglement is preserved better in the hippocampus than in cortex. Figure 5d shows the correlation plots between plaque area percentage and the entanglement parameters: mean linear entropy and mean tangle, analyzed in both the cortex and hippocampus for 5xFAD samples. For plaque area *vs* mean linear entropy, the Pearson’s correlation coefficient (*r*) is −0.88, indicating a strong negative correlation. This suggests that as plaque area increases, mean linear entropy decreases, implying that larger plaque deposits are associated with reduced quantum coherence (or higher decoherence), as reflected by lower linear entropy values. In contrast, for plaque area *vs* mean tangle, the Pearson correlation coefficient (*r*) is 0.91, demonstrating a strong positive correlation. This indicates that as plaque area increases, mean tangle values also rise, suggesting a relationship between greater plaque deposition and higher tangle levels. Large field of view confocal images of the regions analyzed in Figure 5a,5b here are provided in the supplementary document (Figure S7).

**Figure 5:**
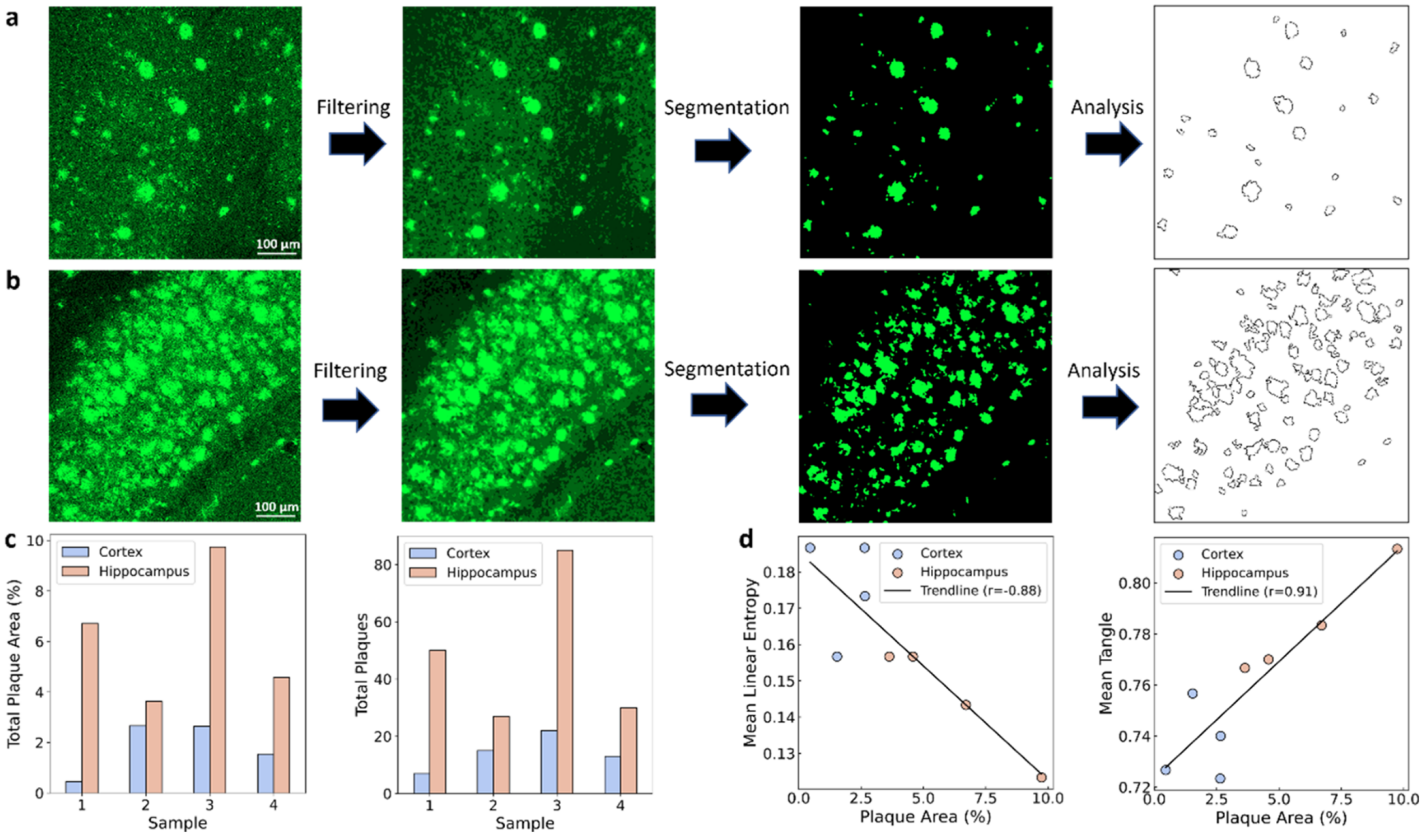
Confocal microscopy validation of 5xFAD mouse brain samples and correlation with quantum parameters. Workflow for analysis of plaque data in the (a) 5xFAD cortex and (b) 5xFAD hippocampus, acquired using a confocal microscope with a field of view of 640 μm × 640 μm. The raw data was filtered and segmented, and the plaque was quantified and analyzed. Amyloid pathology (green) is stained with Alexa Fluor 488. (c) Bar plots displaying the plaque area percentage and total plaque count per sample in the cortex (blue) and hippocampus (orange). (d) Pearson correlation (*r*) plots between the percentage of plaque area and entanglement parameters: mean linear entropy and mean tangle, with trendlines for both the cortex and hippocampus (*r* = −0.88 and *r* = 0.91, respectively). Scale bars: 100 μm.

## Discussion

Through quantum-based sensing, we showed the applicability of entanglement decoherence for distinguishing between amyloidosis and tauopathy models and their respective controls, offering a broader understanding of disease effects on quantum parameters. The intrinsic sensitivity of polarization entangled photons to scattering-induced decoherence enabled clear differentiation between transgenic and control samples, highlighting an inherent quantum advantage over classical polarized-light methods. This study employed a versatile polarization-entangled photon source capable of transforming into different Bell states, achieving shorter integration times and higher photon flux than previous methods. To improve diagnostic accuracy, we incorporated supervised machine learning, which showed robust potential for classifying unseen test samples. Additionally, our findings with the quantum approach were validated through confocal imaging of control samples, establishing consistency between quantum and classical imaging modalities. Together, these results underscore the feasibility of quantum sensing to detect nuanced pathological features in AD.

Interestingly, we observed that P301L and 5xFAD samples preserved entanglement to a distinguishable degree over NTL and CD1 samples, a finding that contrasts with expectations given the transformative nature of the disease. This result may stem from the loss of fine neural structures in AD, leading to a simplified or less complex tissue matrix compared to healthy samples, which are rich in microstructural variability.^35^ These larger structures appear to maintain polarization entanglement more effectively under quantum illumination, while healthy tissue’s microstructural complexity induces more phase disruption, leading to greater entanglement loss. This unique behavior underscores the sensitivity of quantum coherence to structural variations at different scales, presenting a new way to detect AD-related changes at a molecular level.

Quantum sensing exploits intrinsic quantum correlations fundamentally sensitive to scattering-induced decoherence, directly measuring subtle coherence changes that classical polarimetry methods detect only indirectly through intensity or polarization-state changes. This intrinsic quantum sensitivity potentially enables earlier detection of subtle pathological changes in tissues without reliance on staining or external contrast agents, highlighting the clear potential of quantum sensing for advancing label-free, low-intensity biomedical imaging. However, our current demonstration remains an early-stage proof-of-concept. Explicit quantitative comparisons of sensitivity or contrast enhancement relative to classical polarized microscopy were beyond this study’s scope. Additionally, our quantum imaging approach currently operates only in transmission mode using tissue slices, imposing practical limitations related to sample preparation and clinical translation. Significant technological development, including transitioning to reflection-based imaging, remains necessary for direct clinical translation.

Beyond these fundamental considerations, it is important to assess the reproducibility and scalability of the method for larger preclinical studies and eventual clinical translation. Our current study demonstrated measurements in four mouse models (P301L, NTL, 5xFAD, CD1) and across two key brain regions (cortex and hippocampus). In each replicate, tangle and linear entropy data fell on the Werner curve, with diseased and control points forming nonoverlapping clusters. Standard deviations (SD) across repeated measurements per spot and across both regions of interest were low (mean within-spot SD ≤ 0.02 for tangle and ≤ 0.03 for linear entropy), supporting measurement reproducibility. The clear separation across four mouse models suggests the trend will persist in larger cohorts, although systematic studies across broader sample sets will be necessary to fully characterize biological variability across ages, disease stages, and animal strains. Scaling this method to additional brain regions is technically straightforward, but region-specific scattering properties will likely shift absolute tangle and linear entropy coordinates.

Future studies will systematically quantify sensitivity and contrast relative to classical methods to validate quantum sensing’s explicit advantages. To enhance practical feasibility, we aim to extend our technique to reflection-based quantum sensing, potentially enabling *in vivo* detection of Alzheimer’s *via* retinal amyloid-β deposits that are more readily accessible in early-stage Alzheimer’s disease.^36–38^ Utilizing single photons at femto-watt power levels underscores the potential of our approach as a noninvasive, safer diagnostic alternative, paving the way toward clinically relevant quantum-enhanced sensing. For clinical translation, several technical challenges must be addressed alongside potential mitigation strategies. For *in vivo* settings, entangled photons must propagate through scattering tissue without substantial loss of quantum correlations. Employing high-brightness, waveguide-based SPDC sources with high pair generation rates can increase photon throughput, reduce integration times, and improve SNR in thick or optically heterogeneous regions. Photon collection in reflection mode is hampered by backscattering and depolarization, which can be mitigated using high-NA epi-objectives, multimode fibers, high-efficiency detectors (*e.g.*, SNSPDs), and adaptive wavefront control *via* spatial light modulators or digital micromirror devices. Alignment stability is critical for reproducibility, and closed-loop scanning and rotation stages can minimize drift. Transitioning from a bulky tabletop setup to a compact, fiber-coupled platform using integrated photonics would improve portability and ease of use without degrading correlations. Finally, to enhance interpretability and adoption, coregistration with established modalities, such as optical coherence tomography (OCT), would enable precise spatial mapping of quantum measurements to pathology, supported by hardware coalignment for a shared coordinate frame.

## Methods

### Quantum Entanglement Parameters

Photon scattering through biological tissues can degrade quantum correlations, converting initially maximally entangled photon states into partially mixed states. Such mixed states are effectively described by a quantum Werner state model, which represents a controlled mixture between a maximally entangled state and completely uncorrelated noise. To quantify these scattering-induced effects, we reconstruct the quantum state using quantum state tomography and compute relevant entanglement metrics, including tangle, and linear entropy.

Tangle specifically quantifies the degree of entanglement or nonseparability, ranging from 0 for completely separable (non-entangled) states to 1 for maximally entangled states. Tangle was calculated from the concurrence (*C*) as

$$T=C^{2}$$

where *C* = max­(0, λ_1_ – λ_2_ – λ_3_ – λ_4_) and λ*_i_* are the square roots of the eigenvalues of ρ­(σ_*y*_ ⊗ σ_*y*_)­ρ*­(σ_*y*_ ⊗ σ_*y*_) in descending order, with ρ the reconstructed density matrix.

Linear Entropy measures the purity of the quantum state, reflecting its coherence or mixture. It ranges from 0 for a pure (fully coherent) quantum state to 1 for a completely mixed (fully incoherent) state. Linear entropy was computed as

$$S_{L}=\frac{4}{3}\left(1-\mathrm{Tr}\left[\rho^{2}\right]\right)$$

where ρ is the reconstructed density matrix.

Together, these parameters provide complementary insights into how photon scattering affects the quantum correlations necessary for sensitive quantum imaging.

### Animal Models

Transgenic pR5 (B6.Dg-Tg­(Thy1.2-TauP301L)) (P301L) mice have been engineered to express the human 4-repeat tau isoform under the control of the murine Thy1.2 promoter. These mice were backcrossed with C57BL/6J mice for over 20 generations, ensuring a stable C57BL/6J background. For this study, 4 P301L mice of both sexes and their nontransgenic littermates (NTL, *n* = 4), aged 10–12 months, were used. The 5xFAD (B6.Cg-Tg­(APPSwFILon,PSEN1*M146L*L286 V)­6799Vas/Mmjax) transgenic mouse model, commonly used to study Alzheimer’s disease, overexpresses both mutant human amyloid precursor protein (APP) and presenilin-1 (PSEN1), carrying a total of five familial AD (FAD) mutations. These include the Swedish (K670N/M671L), Florida (I716 V), and London (V717I) mutations in APP, as well as the M146L and L286 V mutations in PSEN1. Four 5xFAD mice (aged 5–6 months, both sexes) were also used, along with age-matched CD1 wild-type mice (*n* = 4, both sexes) serving as controls. Animals were housed in ventilated cages inside a temperature-controlled room under specific pathogen-free conditions and under a 12-h dark/light cycle. Pelleted food (3437PXL15, CARGILL) and water were provided ad libitum. Paper tissue and red Tecniplast mouse house (Tecniplast, Italy) shelters were placed in cages for environmental enrichment. All experiments were performed in accordance with the Swiss Federal Act on Animal Protection and were approved by the Cantonal Veterinary Office Zurich.

### Immunofluorescence Staining

P301L and NTL mice were perfused under ketamine/xylazine/acepromazine maleate anesthesia (75/10/2 mg/kg body weight, i.p. bolus injection) with ice-cold 0.1 M phosphate-buffered saline (pH 7.4) and in 4% paraformaldehyde in 0.1 M phosphate-buffered saline (pH 7.4), and fixed for 24 h in 4% paraformaldehyde (pH 7.4) and then stored in 0.1 M PBS (pH 7.4) at 4°C. Coronal brain sections (40 μm) were cut around Bregma 0 to −2 mm and stained with antiphosphorylated tau (pSer202/pThr205) antibody AT-8 (Invitrogen, MN1020, 1:1000). As second we used antibody goat-anti-Rabbit Alexa488 (Invitrogen A11034, 1:200). Sections were counterstained using 4′,6-diamidino-2-phenylindole (DAPI) and mounted with VECTASHIELD antifade fluorescent mounting media (Vector Laboratories H-1000–10).

#### 5xFAD Mice

The mice were transcardially perfused with 30 mL phosphate-buffered saline (PBS), followed by 30 mL 4% paraformaldehyde (PFA) in PBS. Brains were extracted, postfixed in PFA for 24 h, and cryoprotected in 30% sucrose for at least 2 days prior to freezing and sectioning into 40 μm slices. Prior to staining brain sections were pretreated with 70% Formic Acid for 3 min to expose the Aβ epitope. The sections were sequentially washed in 0.1 M PBS (3 times for 10 min), treated with 5% donkey serum in 0.3% PBS with Triton X-100 (PBST) for 30 min followed by overnight incubation at 4°C with the primary antibody, anti-β-Amyloid, 1–16 antibody (6E10,1:1000, SIG-39320, BioLegend). On the second day, the sections were washed for 10 min in 0.3% PBST and incubated for 60 min in 0.3% PBST with 5% donkey serum containing the respective donkey-raised secondary antibody conjugated to Alexa Fluor 488 (1:1000, AB150105, Abcam). After the incubation, the sections were washed again in 0.1 M PBS (3 times for 10 min) and mounted on slides. Finally, the slides were coverslipped with VECTASHIELD (with DAPI) solution.

#### CD1 Mice

The mice were transcardially perfused with 30 mL PBS, followed by 30 mL 4% PFA in PBS. Brains were extracted and postfixation of the brain was performed in PFA for 12–24 h at 4°C. After washing with PBS, the brain was cryoprotected in 30% sucrose solution until fully submerged. Brain sections were then cut into 40 μm slices using a cryostat. For immunohistochemical staining, the sections were incubated in a blocking buffer containing 5% normal donkey serum and 0.3% Triton X-100 in PBS to minimize nonspecific binding. The tissue sections were then incubated overnight at 4°C with the primary antibody against c-FOS (*e.g.*, rabbit anti-c-FOS) diluted in blocking buffer. After washing in PBS, sections were incubated with a fluorescent secondary antibody (*e.g.*, Alexa Fluor 488) for 2 h at room temperature. Following another PBS wash, the sections were mounted on slides using antifade mounting medium.

Although histological staining was performed to independently validate the presence and spatial distribution of Tau tangles and Amyloid-β plaques, it is important to clarify that our quantum sensing measurements do not rely on staining. The optical properties probed by polarization-entangled photons, particularly scattering-induced decoherence, are intrinsic properties of the tissue structure and are unaffected by staining agents. Staining was employed only as an independent validation tool, providing ground-truth biological confirmation. However, future studies may include unstained samples to further emphasize clinical applicability.

### Confocal Microscope

The brain sections were imaged at 10× magnification using a CLSM 900 AiryScan confocal microscope (Zeiss, Germany) with the same acquisition settings applied to all brain slices. Image analysis was performed using Python and ImageJ/FIJI (NIH, USA).

## Supplementary Material



## Data Availability

All study data are included in the article and/or Supporting Information. The custom-made codes of this study are available at GitHub.^39^

## Notes

Reconstructed density matrices of the remaining Bell states as it passes through the transgenic and control brain tissue samples (Figure S1); comparison of the entanglement decoherence of the remaining Bell states as it passes through the transgenic and control samples (Figure S2); large field of view confocal images of 5xFAD mouse brain tissue, highlighting the cortical and hippocampal regions analyzed in Figure 5a,b of the manuscript (Figure S3); spatial decoherence near an Aβ protein aggregate. The confocal image highlights the aggregate, and the corresponding plot shows tangle values as a function of lateral distance across the plaque (Figure S4); representative confocal images from all biological samples in the cortex and hippocampus, respectively, for the four mouse models used in this study (Figures S5 and S6); the comparison of 2D (linear entropy and tangle) and 1D (Werner probability) SVM classification performance for distinguishing 5xFAD from CD1 cortex measurements (Figure S7); complete SVM classifier performance metrics for different test samples in the hippocampal region (Table S1); compares 1D and 2D SVM classifier performance metrics for the data set shown in Figure S7 (Table S2). (PDF)

## References

[1] Scheltens P., Blennow K., Breteler M. M. B., de Strooper B., Frisoni G. B., Salloway S., Van der Flier W. M. (2016). Alzheimer’s
Disease. Lancet.

[2] Rao Y. L., Ganaraja B., Murlimanju B. V., Joy T., Krishnamurthy A., Agrawal A. (2022). Hippocampus and Its
Involvement in Alzheimer’s
Disease: A Review. 3 Biotech.

[3] Wu B.-S., Zhang Y.-R., Li H.-Q., Kuo K., Chen S.-D., Dong Q., Liu Y., Yu J.-T. (2021). Cortical
Structure
and the Risk for Alzheimer’s Disease: A Bidirectional Mendelian
Randomization Study. Transl. Psychiatry.

[4] Sulheim E., WiderØe M., Bäck M., Nilsson K. P. R., Hammarström P., Nilsson L. N. G., de Lange Davies C., Åslund A. K. O. (2023). Contrast
Enhanced Magnetic Resonance Imaging of Amyloid-β Plaques in
a Murine Alzheimer’s Disease Model. J.
Alzheimers Dis..

[5] Chételat G., Arbizu J., Barthel H., Garibotto V., Law I., Morbelli S., van de Giessen E., Agosta F., Barkhof F., Brooks D. J., Carrillo M. C., Dubois B., Fjell A. M., Frisoni G. B., Hansson O., Herholz K., Hutton B. F., Jack C. R., Lammertsma A. A., Landau S. M., Minoshima S., Nobili F., Nordberg A., Ossenkoppele R., Oyen W. J. G., Perani D., Rabinovici G. D., Scheltens P., Villemagne V. L., Zetterberg H., Drzezga A. (2020). Amyloid-PET and 18F-FDG-PET in the Diagnostic Investigation
of Alzheimer’s Disease and Other Dementias. Lancet Neurol..

[6] Kester M. I., Scheffer P. G., Koel-Simmelink M. J., Twaalfhoven H., Verwey N. A., Veerhuis R., Twisk J. W., Bouwman F. H., Blankenstein M. A., Scheltens P., Teunissen C., van der Flier W. M. (2012). Serial CSF Sampling in Alzheimer’s
Disease:
Specific versus Non-Specific Markers. Neurobiol.
Aging.

[7] Roberts B. R., Lind M., Wagen A. Z., Rembach A., Frugier T., Li Q.-X., Ryan T. M., McLean C. A., Doecke J. D., Rowe C. C., Villemagne V. L., Masters C. L. (2017). Biochemically-Defined
Pools of Amyloid-β in Sporadic Alzheimer’s Disease: Correlation
with Amyloid PET. Brain.

[8] Maezawa I., Hong H.-S., Liu R., Wu C.-Y., Cheng R. H., Kung M.-P., Kung H. F., Lam K. S., Oddo S., LaFerla F. M., Jin L.-W. (2008). Congo Red
and Thioflavin-T Analogs
Detect Aβ Oligomers. J. Neurochem..

[9] Lichtenegger A., Muck M., Eugui P., Harper D. J., Augustin M., Leskovar K., Hitzenberger C. K., Woehrer A., Baumann B. (2018). Assessment
of Pathological Features in Alzheimer’s Disease Brain Tissue
with a Large Field-of-View Visible-Light Optical Coherence Microscope. Neurophotonics.

[10] Bolmont T., Bouwens A., Pache C., Dimitrov M., Berclaz C., Villiger M., Wegenast-Braun B. M., Lasser T., Fraering P. C. (2012). Label-Free
Imaging of Cerebral β-Amyloidosis with Extended-Focus Optical
Coherence Microscopy. J. Neurosci..

[11] Baumann B., Woehrer A., Ricken G., Augustin M., Mitter C., Pircher M., Kovacs G. G., Hitzenberger C. K. (2017). Visualization
of Neuritic Plaques in Alzheimer’s Disease by Polarization-Sensitive
Optical Coherence Microscopy. Sci. Rep..

[12] Luo Y., Wang A., Liu M., Lei T., Zhang X., Gao Z., Jiang H., Gong H., Yuan J. (2017). Label-Free Brainwide
Visualization of Senile Plaque Using Cryo-Micro-Optical Sectioning
Tomography. Opt. Lett..

[13] Ji M., Arbel M., Zhang L., Freudiger C. W., Hou S. S., Lin D., Yang X., Bacskai B. J., Xie X. S. (2018). Label-Free Imaging of Amyloid Plaques
in Alzheimer’s
Disease with Stimulated Raman Scattering Microscopy. Sci. Adv..

[14] Wang S., Lin B., Lin G., Sun C., Lin R., Huang J., Tao J., Wang X., Wu Y., Chen L., Chen J. (2019). Label-Free
Multiphoton Imaging of β-Amyloid Plaques in Alzheimer’s
Disease Mouse Models. Neurophotonics.

[15] He C., He H., Chang J., Chen B., Ma H., Booth M. J. (2021). Polarisation
Optics for Biomedical and Clinical Applications: A Review. Light: Sci. Appl..

[16] Tuchin V. V. (2016). Polarized
Light Interaction with Tissues. J. Biomed. Opt..

[17] Borovkova M., Sieryi O., Lopushenko I., Kartashkina N., Pahnke J., Bykov A., Meglinski I., Borovkova M. (2022). Screening of Alzheimer’s Disease With Multiwavelength
Stokes Polarimetry in a Mouse Model. IEEE Trans.
Med. Imaging.

[18] Borovkova M., Bykov A., Popov A., Pierangelo A., Novikova T., Pahnke J., Meglinski I. (2020). Evaluating
β-Amyloidosis Progression in Alzheimer’s Disease with
Mueller Polarimetry. Biomed. Opt. Express.

[19] Pedram A., Besaga V. R., Setzpfandt F., Müstecaplıoğlu Ö. E. (2024). Nonlocality Enhanced Precision in
Quantum Polarimetry via Entangled
Photons. Adv. Quantum Technol..

[20] Mamani S., Shi L., Ahmed T., Karnik R., Rodríguez-Contreras A., Nolan D., Alfano R. (2018). Transmission of Classically Entangled
Beams through Mouse Brain Tissue. J. Biophotonics.

[21] Biton N., Kupferman J., Arnon S. (2021). OAM Light Propagation through Tissue. Sci. Rep..

[22] Shi L., Galvez E. J., Alfano R. R. (2016). Photon Entanglement Through Brain
Tissue. Sci. Rep..

[23] Lib O., Bromberg Y. (2022). Quantum Light in Complex
Media and Its Applications. Nat. Phys..

[24] Lum D. J., Mazurek M. D., Mikhaylov A., Parzuchowski K. M., Wilson R. N., Jimenez R., Gerrits T., Stevens M. J., Cicerone M. T., Camp C. H. (2021). Witnessing
the Survival of Time-Energy
Entanglement through Biological Tissue and Scattering Media. Biomed. Opt. Express.

[25] Varnavski O., Gunthardt C., Rehman A., Luker G. D., Goodson T. I. (2022). Quantum
Light-Enhanced Two-Photon Imaging of Breast Cancer Cells. J. Phys. Chem. Lett..

[26] Mamani S., Shi L., Nolan D., Alfano R. (2019). Majorana Vortex Photons a Form of
Entangled Photons Propagation through Brain Tissue. J. Biophotonics.

[27] Restuccia S., Gibson G. M., Cronin L., Padgett M. J. (2022). Measuring Optical
Activity with Unpolarized Light: Ghost Polarimetry. Phys. Rev. A.

[28] Galvez E. J., Sharma B., Williams F. K., You C.-J., Khajavi B., Castrillon J., Shi L., Mamani S., Sordillo L. A., Zhang L., Alfano R. R. (2022). Decoherence
of Photon Entanglement
by Transmission through Brain Tissue with Alzheimer’s Disease. Biomed. Opt. Express.

[29] Kim T., Fiorentino M., Wong F. N. C. (2006). Phase-Stable Source of Polarization-Entangled
Photons Using a Polarization Sagnac Interferometer. Phys. Rev. A.

[30] Jabir M. V., Samanta G. K. (2017). Robust, High Brightness, Degenerate
Entangled Photon
Source at Room Temperature. Sci. Rep..

[31] Pennanen C., Kivipelto M., Tuomainen S., Hartikainen P., Hänninen T., Laakso M. P., Hallikainen M., Vanhanen M., Nissinen A., Helkala E.-L., Vainio P., Vanninen R., Partanen K., Soininen H. (2004). Hippocampus and Entorhinal
Cortex in Mild Cognitive Impairment and Early AD. Neurobiol. Aging.

[32] Werner R. F. (1989). Quantum
States with Einstein-Podolsky-Rosen Correlations Admitting a Hidden-Variable
Model. Phys. Rev. A.

[33] Zhang Y.-S., Huang Y.-F., Li C.-F., Guo G.-C. (2002). Experimental Preparation
of the Werner State via Spontaneous Parametric Down-Conversion. Phys. Rev. A.

[34] James D. F. V., Kwiat P. G., Munro W. J., White A. G. (2001). Measurement
of Qubits. Phys. Rev. A.

[35] Sabuncu M. R., Desikan R. S., Sepulcre J., Yeo B. T. T., Liu H., Schmansky N. J., Reuter M., Weiner M. W., Buckner R. L., Sperling R. A., Fischl B. (2011). The Dynamics of Cortical and Hippocampal
Atrophy in Alzheimer Disease. Arch. Neurol..

[36] Koronyo-Hamaoui M., Koronyo Y., Ljubimov A. V., Miller C. A., Ko M. K., Black K. L., Schwartz M., Farkas D. L. (2011). Identification of
Amyloid Plaques in Retinas from Alzheimer’s Patients and Noninvasive *in Vivo* Optical Imaging of Retinal Plaques in a Mouse Model. NeuroImage.

[37] Snyder P. J., Alber J., Alt C., Bain L. J., Bouma B. E., Bouwman F. H., DeBuc D. C., Campbell M. C. W., Carrillo M. C., Chew E. Y., Cordeiro M. F., Dueñas M. R., Fernández B. M., Koronyo-Hamaoui M., La Morgia C., Carare R. O., Sadda S. R., van Wijngaarden P., Snyder H. M. (2021). Retinal Imaging in Alzheimer’s and Neurodegenerative
Diseases. Alzheimer’s Dementia.

[38] Qiu Y., Jin T., Mason E., Campbell M. C. W. (2020). Predicting Thioflavin Fluorescence
of Retinal Amyloid Deposits Associated With Alzheimer’s Disease
from Their Polarimetric Properties. Transl.
Vision Sci. Technol..

[39] Shruty19/QuantumSensing_AD_SVM: Repository for code associated with the paper “Discerning Amyloid-β and Tau Pathologies with Learning-Based Quantum Sensing.” This repository contains scripts for SVM-based classification used to distinguish Alzheimer’s disease samples from controls. https://github.com/Shruty19/QuantumSensing_AD_SVM/tree/main. (accessed February 15, 2025).

