# Supplementary material for: Discerning Amyloid‑β and Tau Pathologies with Learning-Based Quantum Sensing
Source: ACS Photonics. 2025 Sep 25;12(10):5510–21. doi: 10.1021/acsphotonics.5c01192 (PMC12532366; doi:10.1021/acsphotonics.5c01192)
Supplement: Supplementary file 1 [file ph5c01192_si_001.pdf]

Supporting Information for

Discerning Amyloid-β and Tau Pathologies with Learning-Based Quantum Sensing

Shruti Sundar[1,2], Marakkarakath Vadakkepurayil Jabir[3], Lukas Glandorf[1,2], Maria Eleni Karakatsani[1,2], Michael Reiss[1,2], Ruiqing Ni[1,2], and Daniel Razansky*[1,2]

[1]Institute for Biomedical Engineering and Institute of Pharmacology and Toxicology, Faculty of Medicine, University of Zurich, Zurich 8057, Switzerland
[2]Institute for Biomedical Engineering, Department of Information Technology and Electrical Engineering, ETH Zurich, Zurich 8093, Switzerland
[3]National Institute of Standards and Technology, Gaithersburg, Maryland 20899, USA

***Corresponding author:** Daniel Razansky
Corresponding author email: daniel.razansky@uzh.ch

This PDF file includes:

Figures S1 to S7
Table S1 to S2

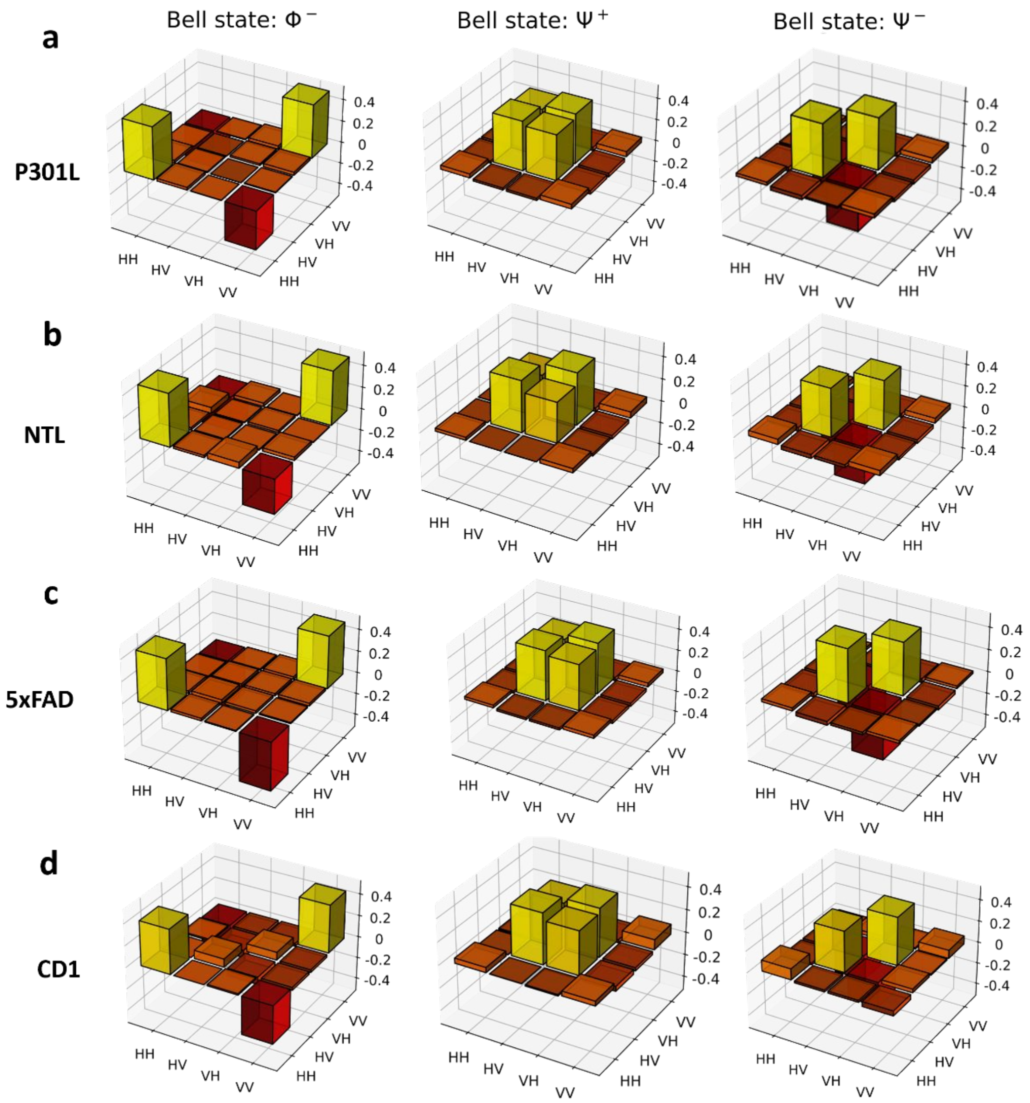

Fig. S1. Reconstructed density matrix of the source after |Φ-⟩, |Ψ+⟩, and |Ψ-⟩ state transmits through (a) P301L , (b) NTL sample (c) 5xFAD, (d) CD1 sample of 40-μm thickness in hippocampus. Experimental results of the tomographic measurements of using different Bell states. Shows the bar-graph of the real part of the reconstructed density matrices of the light. Tomography is performed with coincidence events from 16 set of measurement.

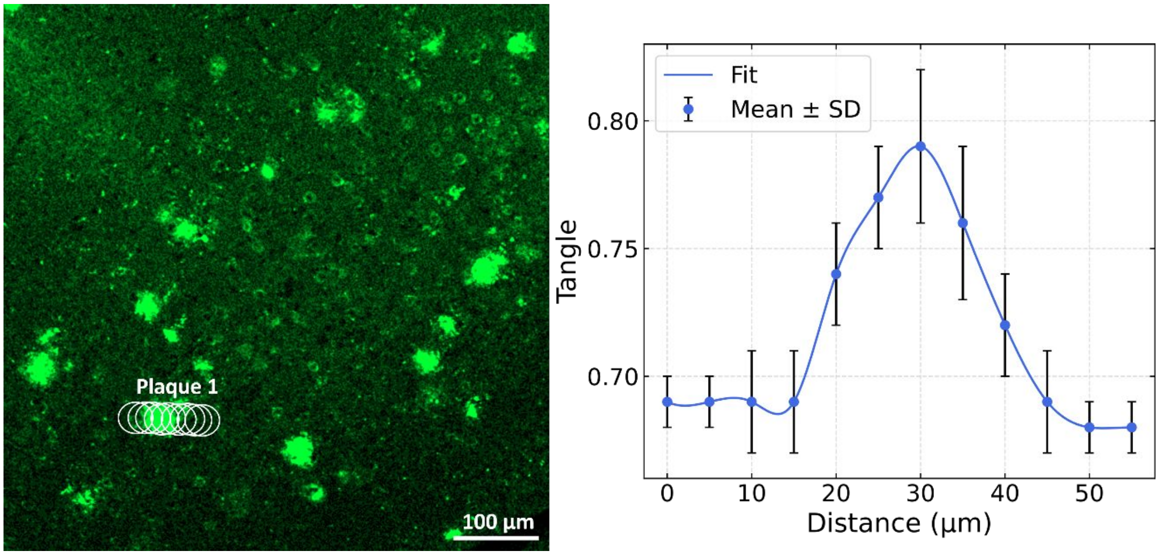

Fig. S2. Spatial decoherence near an Aβ protein aggregate. (a) Confocal image (6E10-stained cortex) from the same field of view showing sparse aggregates. White circles mark measurement spot positions (~20 μm diameter) to illustrate the approximate trajectory of the quantum measurement spot over an aggregate (plaque1). Scale bar: 100 μm. (b) Tangle values plotted as a function of distance. A peak is observed when the beam overlaps the aggregate (30 μm mark), with lower tangle values measured in adjacent regions, demonstrating spatial sensitivity of entanglement to local protein aggregation. Error bars represent standard deviation across repeated measurements.

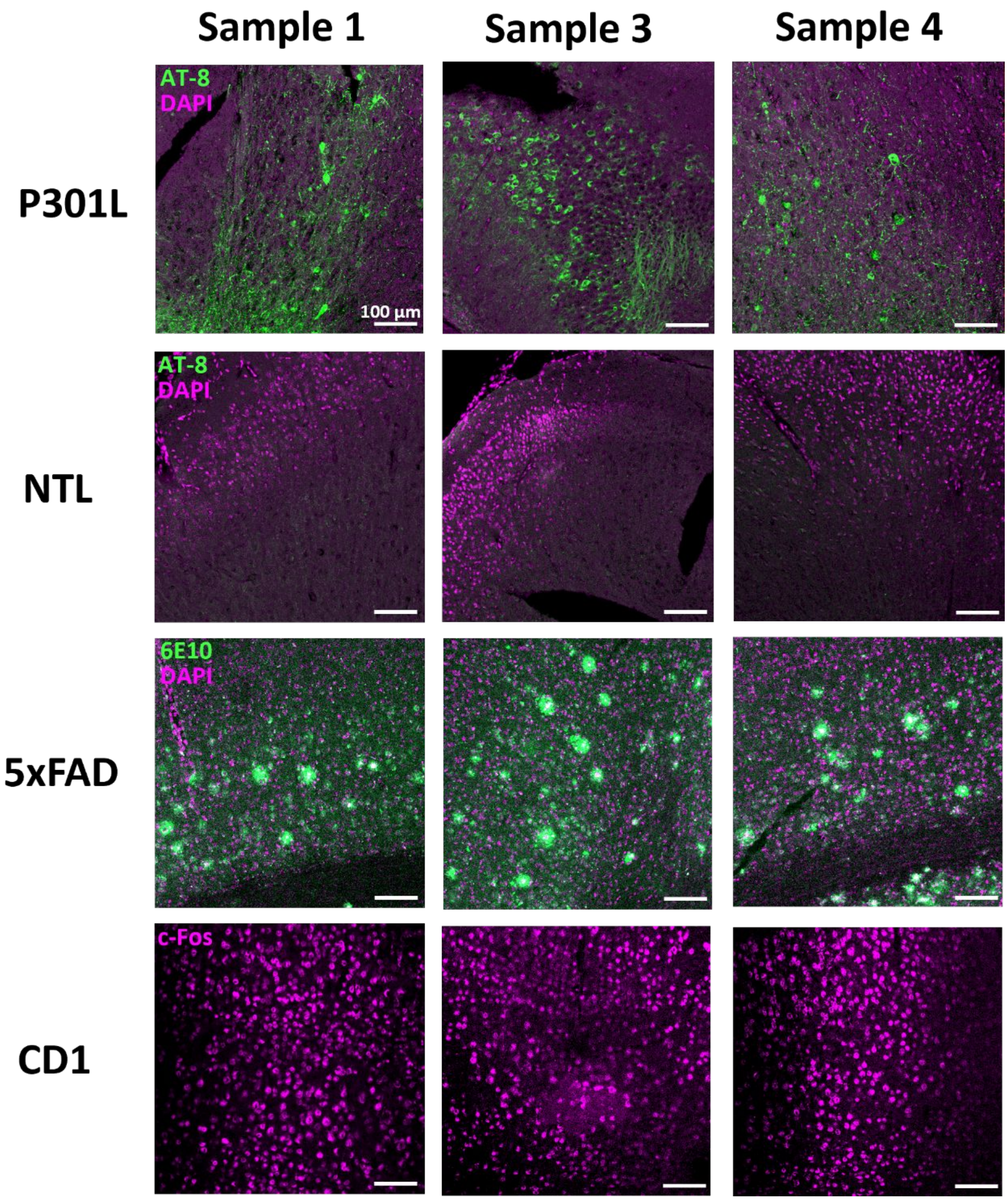

Fig. S3. Confocal fluorescence images of the cortical region from all samples for different mouse models used for histological validation (field-of-view = 640 µm x 640 µm). *First row*: P301L samples stained with anti-phosphorylated tau (AT-8, Alexa Fluor 488, green) and counterstained with DAPI (red). *Second row*: NTL samples processed identically, showing absence of tau pathology. *Third row*: 5xFAD samples stained with anti-amyloid (6E10, Alexa Fluor 488, green) and counterstained with DAPI (red), revealing plaque distribution. *Fourth row*: CD1 samples stained for c-Fos (Alexa Fluor 488, red), indicative of neuronal cell; no Aβ staining was performed on these samples and is expected to be plaque-negative. Scale bars: 100 µm.

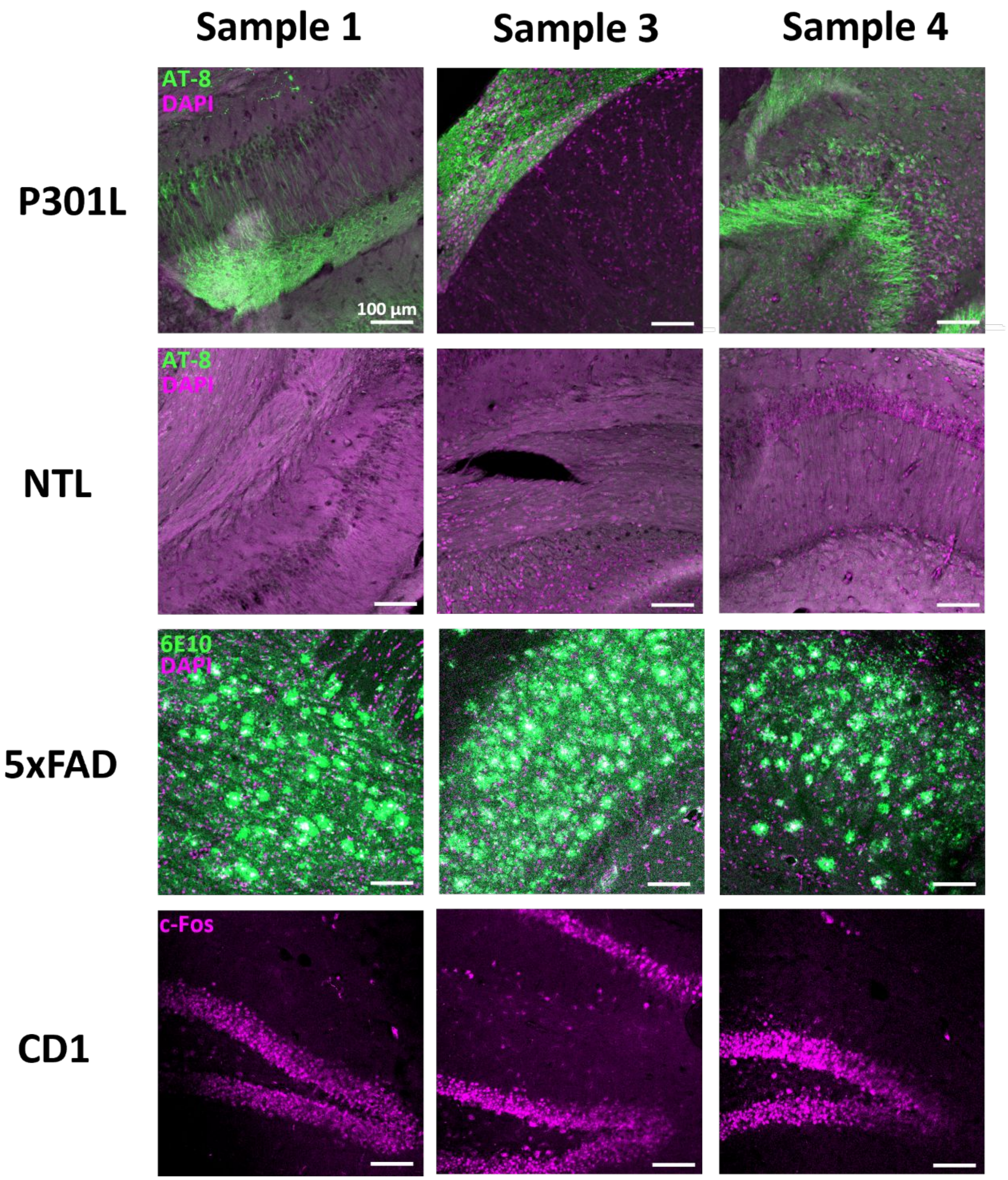

Fig. S4. Confocal fluorescence images of the hippocampal region from all samples for different mouse models used for histological validation (field-of-view = 640 µm × 640 µm). *First row:* P301L samples stained with anti-phosphorylated tau (AT-8, Alexa Fluor 488, green) and counterstained with DAPI (magenta). *Second row:* NTL samples processed identically, showing absence of tau pathology. *Third row:* 5xFAD samples stained with anti-amyloid (6E10, Alexa Fluor 488, green) and counterstained with DAPI (magenta), revealing plaque distribution. *Fourth row:* CD1 samples stained for c-Fos (Alexa Fluor 488, magenta), indicative of neuronal cells; no Aβ staining was performed on these samples and they are expected to be plaque-negative. Scale bars: 100 µm.

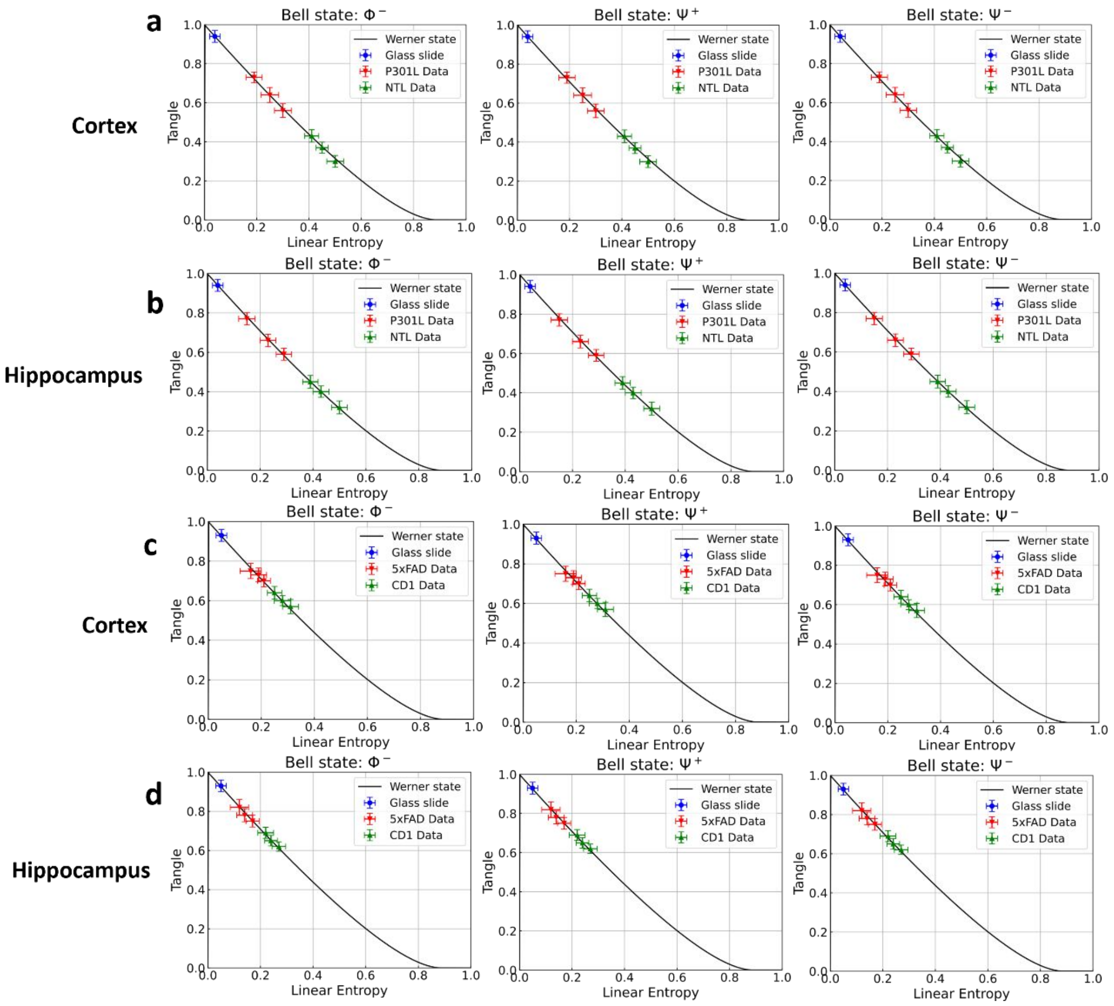

Fig. S5. Comparison of quantum entanglement decoherence across the samples sample using the Werner state model, when measured with the |Φ-⟩, |Ψ+⟩, and |Ψ-⟩ states. Decoherence in P301L and NTL samples measured at (a) cortex and (b) hippocampus. Decoherence in 5xFAD and CD1 samples measured at (c) cortex and (d) hippocampus. Each data point represents a single point scan measured five times in one sample. Error bars indicate the standard deviation of these five measurements per region. The black line represents the theoretical Werner state curve.

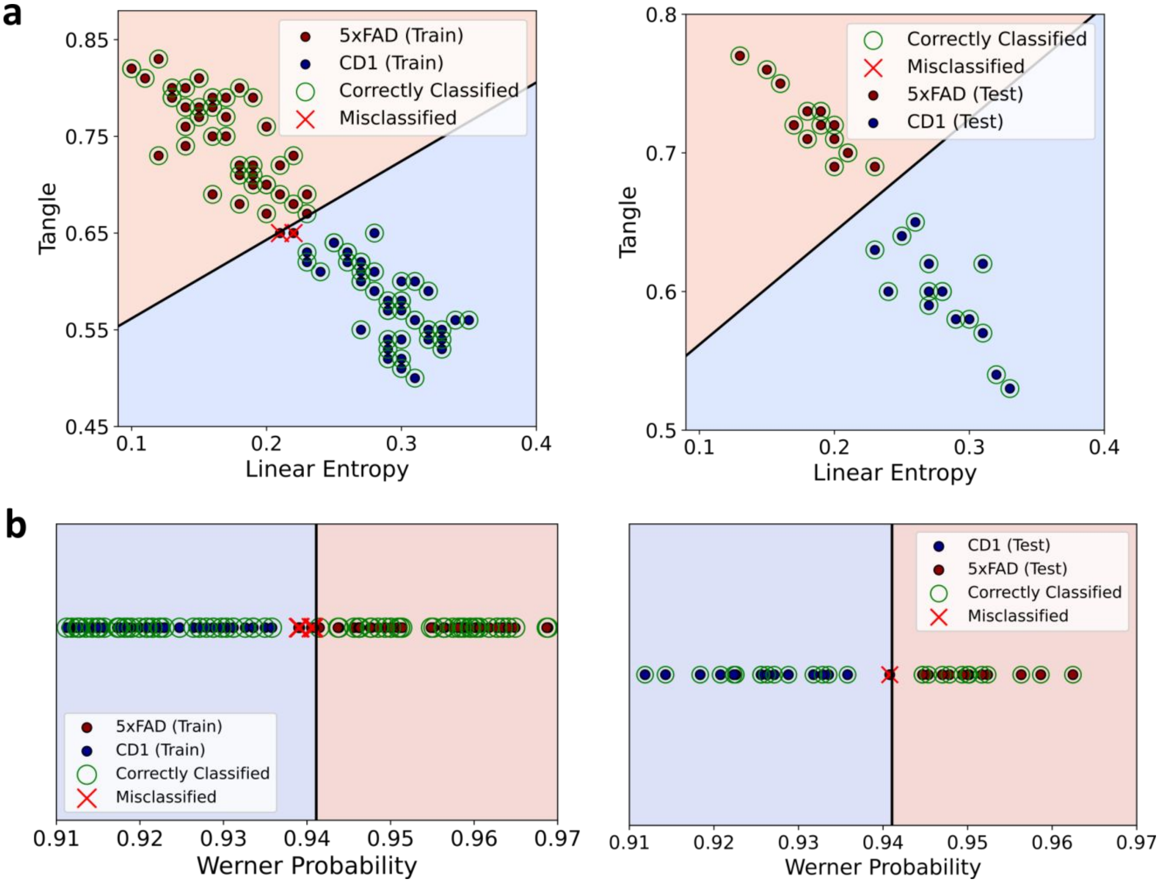

Fig. S6. Comparison of 2D and 1D SVM classification performance for distinguishing 5xFAD from CD1 cortex measurements. (a) 2D SVM using linear entropy and tangle as independent features. Left: training data; right: test data. The decision boundary (black line) separates amyloidosis model (5xFAD, red) from control (CD1, blue). Correctly classified points are outlined in green; misclassified points are marked with red crosses.(b) 1D SVM using Werner probability derived from the same measurements. Left: training data; right: test data. The decision boundary (black vertical line) separates 5xFAD (red) from CD1 (blue). Correct classifications are outlined in green; misclassifications are marked with red crosses.

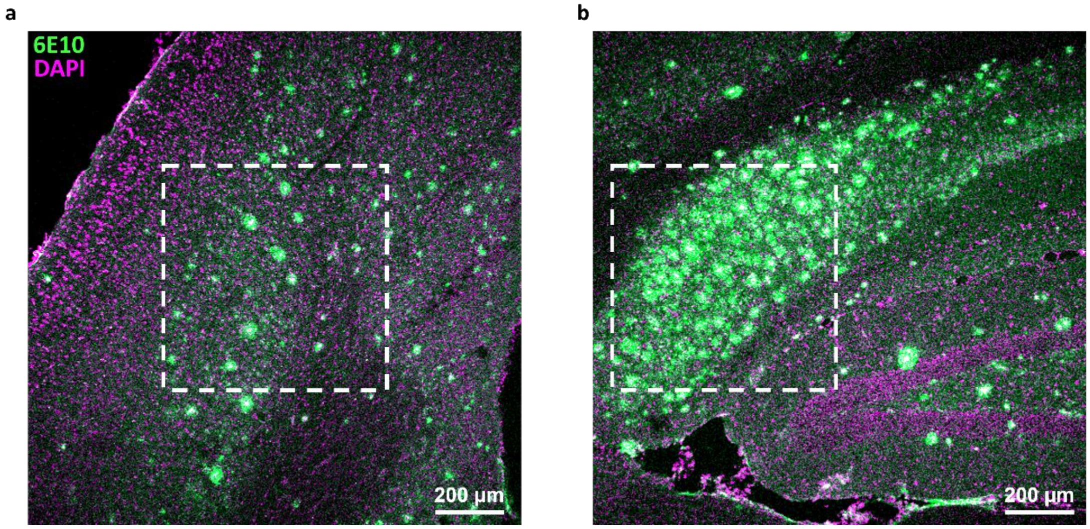

Fig. S7. Large field-of-view (1.42 mm x 1.42 mm) confocal images of 5xFAD mouse brain tissue showing (a) the cortex and (b) the hippocampus. Amyloid-beta plaques (green) were stained using Alexa Fluor 488 conjugated secondary antibody against anti-β-Amyloid (6E10), and nuclei (magenta) were stained using DAPI. The white dotted boxes indicate the regions analysed for plaque, corresponding to the 5xFAD cortex and hippocampus regions in the main Fig. 5a and Fig. 5b, respectively.

Table S1. Complete SVM classifier performance metrics for different test samples in the hippocampal region

Test sample	Region	Precision	Recall	Sensitivity	Specificity	F1-score
CD1: **S1**	Hippocampus	1	0.93	-	0.93	0.97
5xFAD: **S1**		0.94	1	1	-	0.97
CD1: **S2**		1	1	-	1	1
5xFAD: **S2**		1	1	1	-	1
CD1: **S3**		1	1	-	1	1
5xFAD: **S3**		1	1	1	-	1
CD1: **S4**		0.88	1	-	1	0.94
5xFAD: **S4**		1	0.87	0.87	-	0.93
**Mean**	**-**	**0.97**	**0.97**	**0.97**	**0.98**	**0.97**

Table S2. 1D SVM v/s 2D SVM classifier performance metrics for the same dataset.

Test sample	SVM	Cross validation mean	Precision	Recall	Sensitivity	Specificity	F1-score
CD1: **S1**	1D	0.37	0.94	1	-	1	0.97
5xFAD: **S1**			1	0.93	0.93	-	0.97
CD1: **S1**	2D	0.97	1	1	-	1	1
5xFAD: **S1**			1	1	1	-	1
